# The Exosome Cofactor Rrp47 Is Critical for the Stability and Normal Expression of Its Associated Exoribonuclease Rrp6 in *Saccharomyces cerevisiae*

**DOI:** 10.1371/journal.pone.0080752

**Published:** 2013-11-05

**Authors:** Monika Feigenbutz, William Garland, Martin Turner, Phil Mitchell

**Affiliations:** Molecular Biology and Biotechnology Department, The University of Sheffield, Sheffield, United Kingdom; Univ. of Edinburgh, United Kingdom

## Abstract

Rrp6 is a conserved catalytic subunit of the eukaryotic nuclear exosome ribonuclease complex that functions in the productive 3’ end maturation of stable RNAs, the degradation of transiently expressed noncoding transcripts and in discard pathways that eradicate the cell of incorrectly processed or assembled RNAs. The function of Rrp6 in these pathways is at least partially dependent upon its interaction with a small nuclear protein called Rrp47/Lrp1, but the underlying mechanism(s) by which Rrp47 functions in concert with Rrp6 are not established. Previous work on yeast grown in rich medium has suggested that Rrp6 expression is not markedly reduced in strains lacking Rrp47. Here we show that Rrp6 expression in *rrp47∆* mutants is substantially reduced during growth in minimal medium through effects on both transcript levels and protein stability. Exogenous expression of Rrp6 enables normal levels to be attained in *rrp47∆* mutants. Strikingly, exogenous expression of Rrp6 suppresses many, but not all, of the RNA processing and maturation defects observed in an *rrp47∆* mutant and complements the synthetic lethality of *rrp47∆ mpp6∆* and *rrp47∆ rex1∆* double mutants. Increased Rrp6 expression in the resultant *rrp47∆ rex1∆* double mutant suppresses the defect in the 3’ maturation of box C/D snoRNAs. In contrast, increased Rrp6 expression in the *rrp47∆ mpp6∆* double mutant diminishes the block in the turnover of CUTs and in the degradation of the substrates of RNA discard pathways. These results demonstrate that a principal function of Rrp47 is to facilitate appropriate expression levels of Rrp6 and support the conclusion that the Rrp6/Rrp47 complex and Rex1 provide redundant exonuclease activities for the 3’ end maturation of box C/D snoRNAs.

## Introduction

Ribonucleases are of fundamental importance for the expression of both coding and non-coding RNA in all cells. All characterised RNA transcripts are generated from longer precursor molecules through processing reactions involving the nuclease activities of exo- and/or endonucleases. Furthermore, the large amount of RNA fragments that are released as by-products of such processing reactions, such as pre-mRNA introns, must be degraded. The ultimate degradation of mRNA in the cytoplasm is also an essential biological process, individual mRNAs being degraded at transcript-specific rates that contribute to the expression levels of each gene [1]. Furthermore, both coding and non-coding RNAs are subjected to quality control systems that degrade incorrectly processed or assembled ribonucleoprotein (RNP) particles [2]. There is a substantial flux through such RNA surveillance pathways, even in normal healthy cells [3,4].

A major source of 3’ →5’ exoribonuclease activity in eukaryotic cells is the exosome RNase complex, which plays key roles in both the productive 3’ end processing of precursor transcripts to their mature RNAs, and in the complete degradation of RNAs that are targeted to RNA discard pathways [5]. The exosome was initially identified as a nuclease complex that functions in the 3’ end maturation of 5.8S rRNA, snoRNAs and snRNAs [6,7] and subsequently shown to function in cytoplasmic mRNA turnover, and in nuclear and cytoplasmic RNA surveillance pathways for both coding and non-coding RNAs. In addition, the analysis of RNA from yeast strains compromised in exosome activity allowed the discovery of a new class of low abundance RNAs known as cryptic unstable transcripts (CUTs) [8-10]. 

The exosome has two associated catalytic subunits, Rrp44 (also known as Dis3) and Rrp6. Yeast and mammalian Rrp44 is found exclusively associated with the exosome complex [11,12]. Rrp44 belongs to the RNase R/RNase II family of exoribonucleases [13] but is restricted to eukaryotes and contains an additional N-terminal PIN domain that has endonuclease activity [14-16]. Rrp44 has a highly processive hydrolytic exonuclease activity when expressed as a recombinant protein [6] but shows a largely reduced activity when associated with the exosome core complex [17]. Interaction with the exosome is through the N-terminal PIN domain of Rrp44 and the exosome core subunits Rrp41 and Rrp45 [16,18]. The core of the exosome structure functions to channel the RNA substrate to the active site of the Rrp44 exonuclease [19]. Rrp6 is related to the RNase D family of exonucleases [20] that have a “DEDD” catalytic domain named after four highly conserved acidic residues that coordinate the binding of two metal cations required for catalysis [21,22]. The *RRP6* gene was originally cloned by complementation of a catalytically inactive allele (*rrp6-1*) that contains an asparagine in place of the conserved D238 residue [23,24]. In addition to the catalytic domain, Rrp6 also has an N-terminal PMC2NT domain, a central HRDC domain and a C-terminal region that is required for its association with the exosome. Loss of interaction with the exosome has little effect on the ability of Rrp6 to function in RNA processing or RNA degradation pathways [25]. However, association of Rrp6 with the exosome allosterically stimulates the activity of Rrp44 [19,26].

RNA analyses have revealed that most Rrp6-mediated RNA processing and degradation pathways are impeded in strains lacking the nuclear RNA-binding protein Rrp47 (also known as Lrp1) [11]. Rrp47 directly interacts with the PMC2NT domain of Rrp6 through its N-terminal Sas10/C1D domain, while the C-terminal region of the protein is required for RNA binding activity and contributes to substrate recognition [27,28]. Another RNA-binding protein, Mpp6, interacts with exosome complexes and has been proposed to stimulate the activity of Rrp44 [29] or to promote the functional coupling between Rrp6 and the TRAMP/exosome complexes [30]. Strains lacking Rrp6 or Rrp47 are synthetic lethal with *mpp6∆* mutants, probably reflecting a degree of functional redundancy between the Rrp6 and Rrp44 enzymes [3,31]. Similarly, *rrp6∆* and *rrp47∆* mutants are also synthetic lethal with mutants lacking Rex1, another RNase D-related 3’→5’ exoribonuclease [32,33]. 

Cellular ribonucleases represent effective modulators of changes in gene expression profiles. However, little data is available concerning how these enzymes might be regulated in response to changes in physiological conditions or as a result of developmental programmes. Rrp6 expression in diploid yeast is decreased upon shift from fermentation to respiration, and further depleted upon entry into meiosis. This fluctuation of Rrp6 expression occurs without a significant alteration in *RRP6* mRNA levels, indicative of a post-transcriptional mode of regulation [34]. Furthermore, both the *RRP6* and *RRP47* genes are potentially regulated by transcription factors that modulate gene expression in response to nutrient availability or stress [35-37]. In prokaryotes, the 3’→5’ exoribonucleases RNase II and RNase R are both regulated in response to nutrient availability at the level of protein stability. Notably, RNase II protein levels are decreased upon shift from rich medium to minimal medium in a manner dependent upon the protein Gmr [38]. In contrast, RNase R is a highly unstable protein during growth in rich medium and its expression is induced by protein stabilisation upon entry into the stationary phase or upon cold shock [39]. RNase R instability during rapid growth is mediated by acetylation and involves its interaction with the SmpB/tmRNA trans-translation complex [40,41]. The SmpB mRNA accumulates in the absence of RNase R, indicative of a mutually dependent regulation of expression [42]. 

We have recently reported that the absence of Rrp6 has a profound effect on the stability of its associated protein Rrp47, without a substantial change in transcript levels [43]. Previous studies on cultures in rich medium suggested Rrp6 expression levels are not markedly affected in strains lacking Rrp47 [11,27]. Here we report that Rrp6 levels are decreased substantially in the absence of Rrp47 during growth in minimal medium, reflecting both a decrease in protein stability and *RRP6* transcript abundance. Overexpression of Rrp6 suppressed RNA processing and degradation defects observed in the *rrp47∆* mutant and complemented the synthetic lethality of *rrp47∆ mpp6∆* and *rrp47∆ rex1∆* double mutants. Furthermore, analyses of RNA from the *rrp47∆ mpp6∆* and *rrp47∆ rex1∆* double mutants are consistent with studies proposing that either the Rrp6/Rrp47 complex or an Mpp6-dependent activity is required for RNA surveillance pathways and the degradation of CUTs, while the Rrp6/Rrp47 complex and Rex1 provide redundant activities for the 3’ maturation of box C/D snoRNAs [29,44].

## Materials and Methods

### Plasmids

The plasmid expressing an N-terminal Rrp6 fusion protein (zz-Rrp6) with two copies of the z domain of protein A from *Staphylococcus aureus* (p263) has been described previously [45]. This construct expresses the Rrp6 fusion protein from the *RRP4* promoter. An analogous *MPP6* construct has been recently reported [44]. Mutant variants of the *RRP6* construct that express the catalytically inactive *rrp6-1* (D238N) derivative (p389) [24] or just the N-terminal domain truncation (L197X) (p287) were generated by site directed mutagenesis with appropriate primers [43]. A genomic clone of the *RRP6* gene encompassing approximately 400 nucleotides up- and downstream of the ORF (p436) was constructed by amplification of the *RRP6* locus from wild-type genomic DNA by PCR using Vent DNA polymerase and primers o457 (cagtctagacttcgagatgagcttg) and o458 (gctgggcccacctcagtattacagc), and cloning into pRS416 (*URA3* marker) as an *Xba*I-*Eco*RI fragment [46] (the *Eco*RI site is genomically encoded). The *RRP6* promoter region contains the *CEN* element of chromosome 15. To prevent recombination of plasmids containing the genomic *RRP6* sequence during growth in yeast due to the presence of two *CEN* elements, the *CEN6* element within the vector backbone was deleted by site-directed mutagenesis. *Hpa*I sites were introduced either side of the *CEN6* element using the sense primers o839 (gttggcgatccccctagagtcgttaacatcttcggaaaacaaaaactat) and o841 (aattatttttatagcacgtgatgttaacgacccaggtggcacttttcgg) and the intervening sequence was deleted by restriction digestion and religation. A genomic clone of the *RRP47* gene [28] was generated by PCR amplification of wild-type genomic DNA using the primers o191 (aaactcgaggaactgactactga) and o192 (aaagagctcaaactttcgctgg), and the product was cloned into pRS416 as a *Xho*I-*Sac*I fragment. High copy number derivatives of these plasmids were generated by subcloning the inserts into the 2 micron plasmids pRS424 (*TRP1* marker), pRS425 (*LEU2* marker) and pRS426 (*URA3* marker) [47] using appropriate restriction enzymes. *RRP6* and *RRP47* alleles were also subcloned into pRS314 (*TRP1* marker) for plasmid shuffle assays in the *rex1∆ rrp47∆* strain, and into pRS313 (*HIS3* marker) or pRS415 (*LEU2* marker) for plasmid shuffle assays in the *mpp6∆ rrp47∆* strain.

### Strains

Strains were grown at 30 ° C in YPD medium (2 % glucose, 2 % bactopeptone, 1 % yeast extract) or in selective minimal growth medium, comprising 2 % glucose, 0.5 % ammonium sulphate, 0.17 % yeast nitrogen base and the appropriate amino acids and bases. Plasmid shuffle assays were performed on complete minimal medium containing 50 μg/ml uracil and 1 mg/ml 5-flouro-orotic acid (5 FOA) (Melford Laboratories). Colonies recovered from 5 FOA plates were streaked out on appropriate selective solid medium and shown to be cured of the parental *RRP47* or *MPP6* plasmids by lack of growth on SD medium lacking uracil. 

For spot growth assays, precultures were diluted to a standard OD at 600 nm and then 10-fold serially diluted with fresh medium. Aliquots were applied to the surface of minimal medium plates and incubated at 30 °C for 3 days. Cells were harvested from liquid medium cultures at OD at 600 nm of less than 1.0 for protein analyses, or less than 0.5 for RNA analyses.

Strains expressing the C-terminal Rrp47-zz fusion protein, with or without the *rrp6∆::TRP1* allele, have been described previously [11]. Yeast *rrp47∆::KANMX4*, *mpp6∆::KANMX4* and *rex1∆::KANMX4* deletion strains were obtained from Euroscarf (University of Frankfurt, Germany). The *rrp47∆::KANMX4* allele was introduced into the *rrp6-TAP::HIS3* strain (Thermo Fisher Scientific) by PCR-mediated homologous recombination, as described [43]. The *mpp6∆::KANMX4 rrp47::hphMX4* double mutant was made by converting the *KANMX4* marker in the *rrp47::KANMX4* strain to the *hphMX4* marker, using the plasmid pAG32 [48], and then targeting the *RRP47* locus of the *mpp6∆* strain by PCR-mediated integration after transformation with a plasmid encoding a functional *MPP6* gene. The *rex1∆::KANMX4 rrp47∆::KANMX4* double mutant strain has been described previously [28] and was made by crossing *rex1∆* and *rrp47∆* single mutants, transforming the diploid strain with a plasmid encoding a wild-type copy of the *RRP47* gene, and isolating meiotic progeny bearing both null alleles. Strains expressing the plasmid-borne zz-Rrp6 fusion protein as the sole form of the protein and lacking either the *MPP6* or *REX1* gene have been recently reported [44]. 

### Protein Analyses

Cell extracts were prepared under alkaline denaturing conditions to minimise protein degradation [49]. Translational shut-off experiments were performed by addition of cycloheximide to a final concentration of 100 μg/ml and aliquots of the culture were harvested at 10 minute intervals thereafter. Lysates were resolved by SDS-PAGE and the proteins transferred to Hybond C membranes (GE Healthcare) for western analyses. An Rrp6-specific polyclonal antiserum was kindly provided by David Tollervey [11]. Pgk1 was used as a loading control and was detected with a mouse monoclonal antibody (clone 22C5D8, Life Technologies). TAP-tagged and zz fusion proteins were detected using the PAP antibody (P1291, Sigma). For the analysis of non-tagged proteins, blots were incubated with either goat anti-rabbit (A4914, Sigma) or goat anti-mouse (1706516, BioRad) HRP-conjugated secondary antibodies. ECL images were captured and quantified using a G:Box iChemi XL system (Syngene). Expression levels of Rrp6 and Rrp47 proteins were determined relative to the amount of Pgk1 detected on the identical blot for a minimum of 4 independent biological replicates.

### RNA analyses

Total cellular RNA was isolated from cell pellets by glass bead extraction in the presence of phenol and guanidinium isothiocyanate solution, followed by phenol/chloroform extraction and ethanol precipitation [50]. RNA was resolved through 8 % polyacrylamide gels containing 50 % urea and transferred to Hybond N^+^ membranes (GE Healthcare). Northern blots were hybridised at 37 °C with 5’ ^32^P-labelled oligonucleotide probes in 6 x SSPE buffer, 5 x Denhardt’s solution and 0.2 % SDS. The sequences of the oligonucleotide probes used were as follows: U14, tcactcagacatcctagg (o238); snR38, gagaggttacctattattacccattcagacagggataactg (o272); snR13, caccgttactgatttggc (o240); *SCR1*, aaggacccagaactaccttg (o242); U6, atctctgtattgtttcaaattgaccaa (o517); U3, ttcggtttctcactctggggtac (o443); 5.8S, gcgttgttcatcgatgc (o221); *NEL025c*, ggcttctacagaacaagttgtatcgaaatgattgttggcgac (o809); 5S, ctactcggtcaggctc (o925); *IGS1-R*, gatgtaagagacaagtgaacagtgaacagtgaacagtggggaca (o815). Hybridised blots were placed under phosphor storage screens and analysed using a personal molecular imager FX scanner (Biorad). Figures were generated from nonsaturated images using ImageJ64 (NIH, Bethesda).

For cDNA synthesis reactions, RNA samples were cleaned up using RNeasy miniprep kits (Qiagen) and their integrity confirmed by analysis on glyoxal agarose gels. Reverse transcription reactions were performed on DNase I treated RNA, using random hexamer primers with the Tetro cDNA synthesis kit (BioLine). Quantitative real time PCR (qPCR) primers were designed using Primer3Plus software [51] and their specificity confirmed by melt curve analyses and analytical PCR reactions. The qPCR primers used in this study were as follows: *RRP6*(+), tggcttcagcgagatttagg (o650); *RRP6*(-), gcggtcttatacgccagtca (o651); *SCR1*(+), gagagtccgttctgaagtgtcc (o654); *SCR1*(-), cctaaggacccagaactaccttg (o655). Triplicate qPCR reactions were performed on 4 biological replicates in a Corbett Rotor-Gene cycler (Qiagen) using SensiMix SYBR kits (Bioline). Assays were analysed using RotorGene 6000 software and *RRP6* mRNA levels were normalized to the *SCR1* reference transcript using the comparative C_T_ method [52].

## Results

### Rrp6 expression levels are decreased in *rrp47∆* strains

We recently showed that the expression of Rrp47 is strongly dependent upon its ability to interact with Rrp6 and form the Rrp6/Rrp47 complex [43]. Previous work had shown that the lack of Rrp47 does not have a significant impact on the expression level of Rrp6 [11,27] but these earlier studies were nonquantitative and limited to analyses of cultures in rich YPD medium. We therefore reanalysed the relative expression level of Rrp6 in wild-type strains and *rrp47∆* mutants by quantitative western blotting during growth in rich medium (YPD) and in complete minimal medium (SD). Cell lysates were prepared under alkaline denaturing conditions to minimise protein degradation *in vitro* [49].

**Figure 1:**
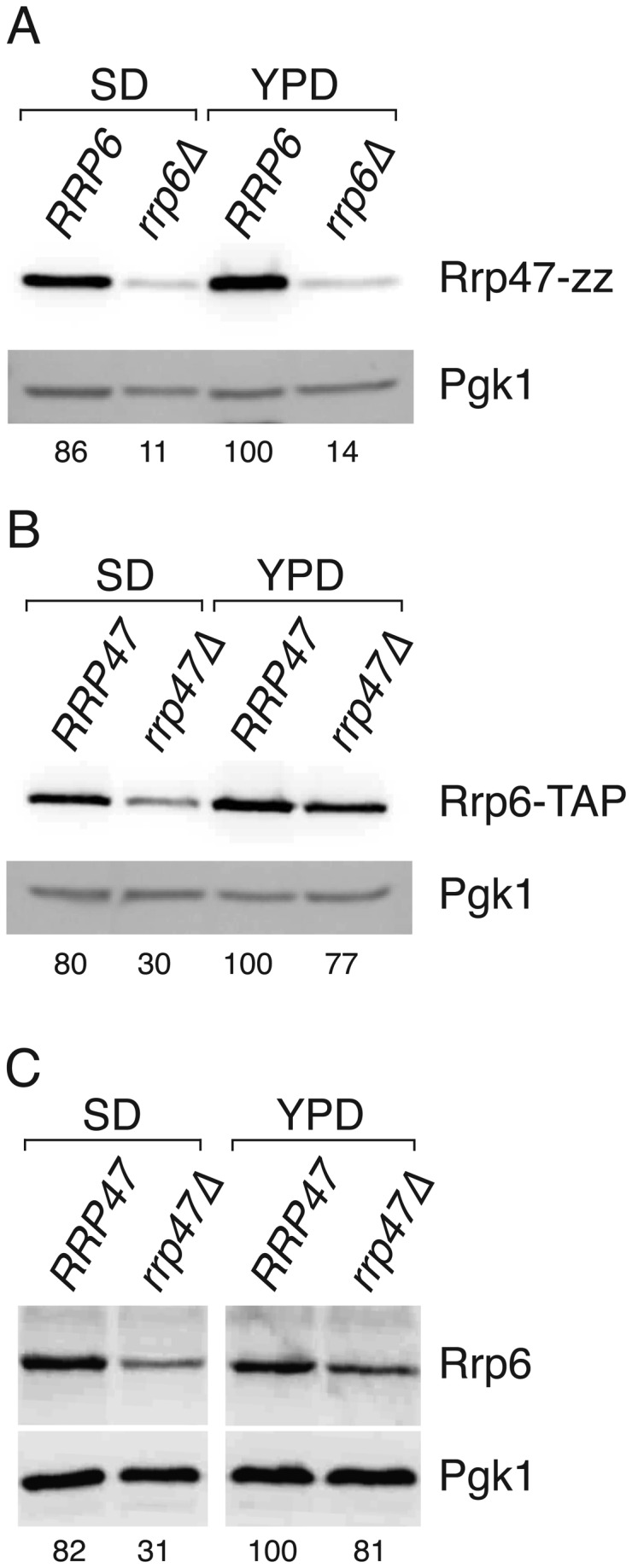
The Expression of Rrp6 and Rrp47 is mutually dependent. Isogenic wild-type and *rrp6∆* or *rrp47∆* strains were grown in selective minimal medium (SD) or in nonselective rich medium (YPD) and extracts were prepared under alkaline denaturing conditions. Extracts were resolved by SDS-PAGE and western blots were incubated with PAP antibody (Panels A and B) to detect fusion proteins, or with an Rrp6-specific antibody (Panel C). Blots were also incubated with an antibody specific to detect Pgk1, which serves as a loading control. (A) Western analysis of Rrp47-zz in isogenic wild-type *RRP6* and *rrp6∆* strains. (B) Western analysis of Rrp6-TAP in isogenic wild-type *RRP47* and *rrp47∆* strains. (C) Western analysis of non-tagged Rrp6 in isogenic wild-type *RRP47* and *rrp47∆* strains. Relative expression levels of Rrp6 or Rrp47, indicated as percentages under each panel, are normalised for Pgk1 expression levels and standardised to the amount of protein in the wild-type strain grown in YPD. Values are the mean of at least 4 independent experiments.

As previously reported [43], the expression level of the Rrp47-zz fusion protein was considerably less in the *rrp6∆* mutant than in the wild-type strain (Figure 1A). In the reciprocal experiment, Rrp6-TAP expression levels in the *rrp47∆* mutant were ~ 80 % of that observed in the wild-type strain during growth in YPD medium (Figure 1B) but were reduced more than two-fold during growth in minimal medium (Figure 1B). The reduction in Rrp6 expression levels in the *rrp47∆* mutant was independent of the TAP tag fusion, since comparable reductions in Rrp6 levels were observed for the Rrp6-TAP fusion protein and for non-tagged, wild-type Rrp6 protein (Figure 1B and C). These data clearly demonstrate that Rrp6 levels are reduced in the absence of Rrp47, and that this effect is responsive to alterations in growth medium. The expression levels of Rrp6 and Rrp47 are mutually dependent, with Rrp47 being more sensitive than Rrp6 to the absence of its partner protein.

**Figure 2:**
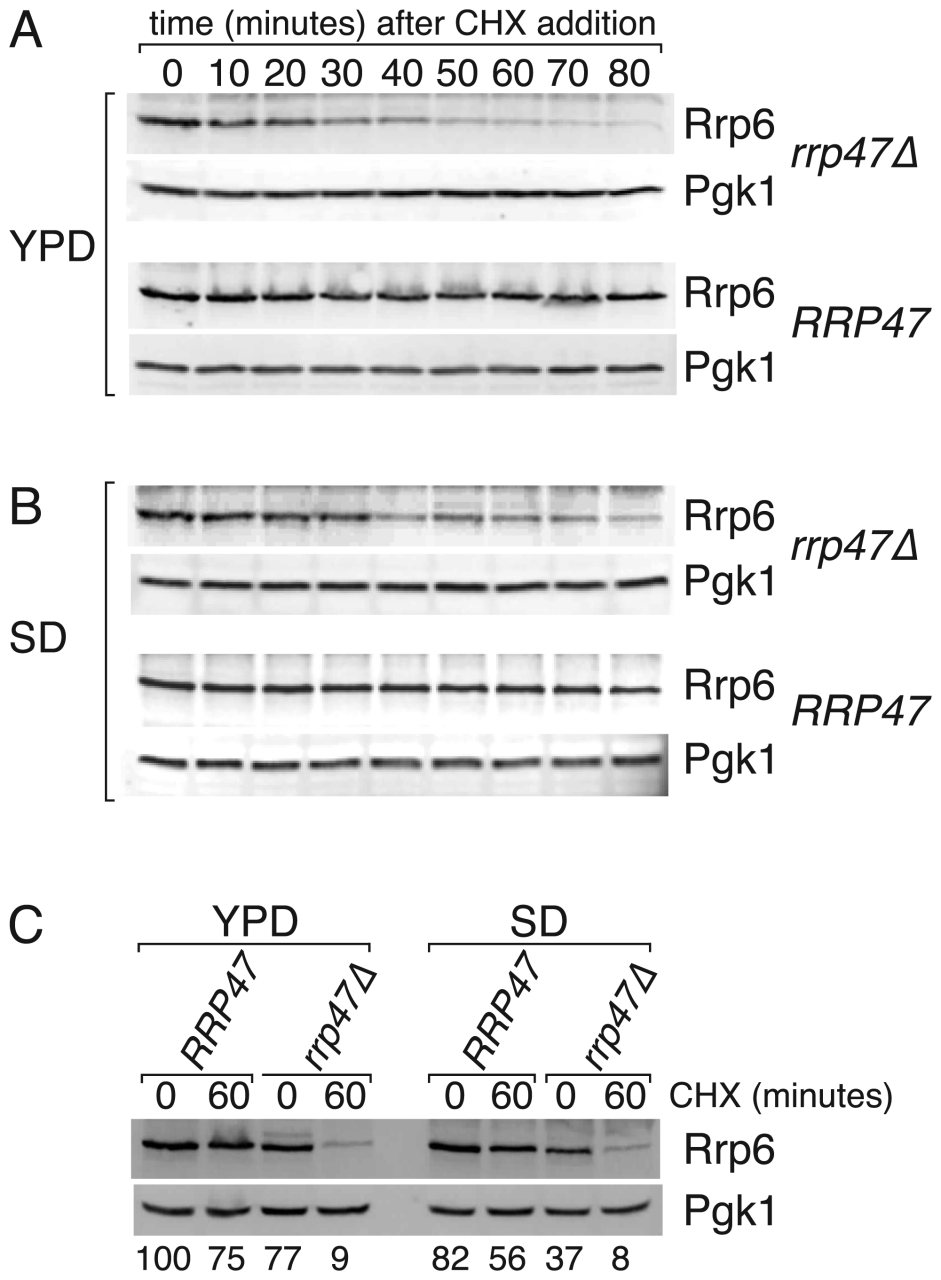
Rrp6 protein stability is decreased in the *rrp47∆* mutant. Isogenic wild-type and *rrp47∆* strains were harvested during growth in selective minimal medium (SD) or rich medium (YPD) and at time-points after addition of the translation inhibitor cycloheximide (CHX), as indicated. Extracts were prepared under denaturing conditions and identical western blots were incubated with antiserum specific to Rrp6 and the loading control Pgk1. (A) Translational shut-off experiment in YPD medium. (B) Translational shut-off experiment in SD medium. (C) Quantitative analysis of the amount of Rrp6 in extracts from wild-type and *rrp47∆* strains before addition of cycloheximide (“0” lanes) and 60 minutes after treatment (“60” lanes). The relative amount of Rrp6, normalised to Pgk1 expression levels and standardised to the level observed in the wild-type strain during growth in YPD medium (average of 2 experiments), is given below each lane.

**Figure 3:**
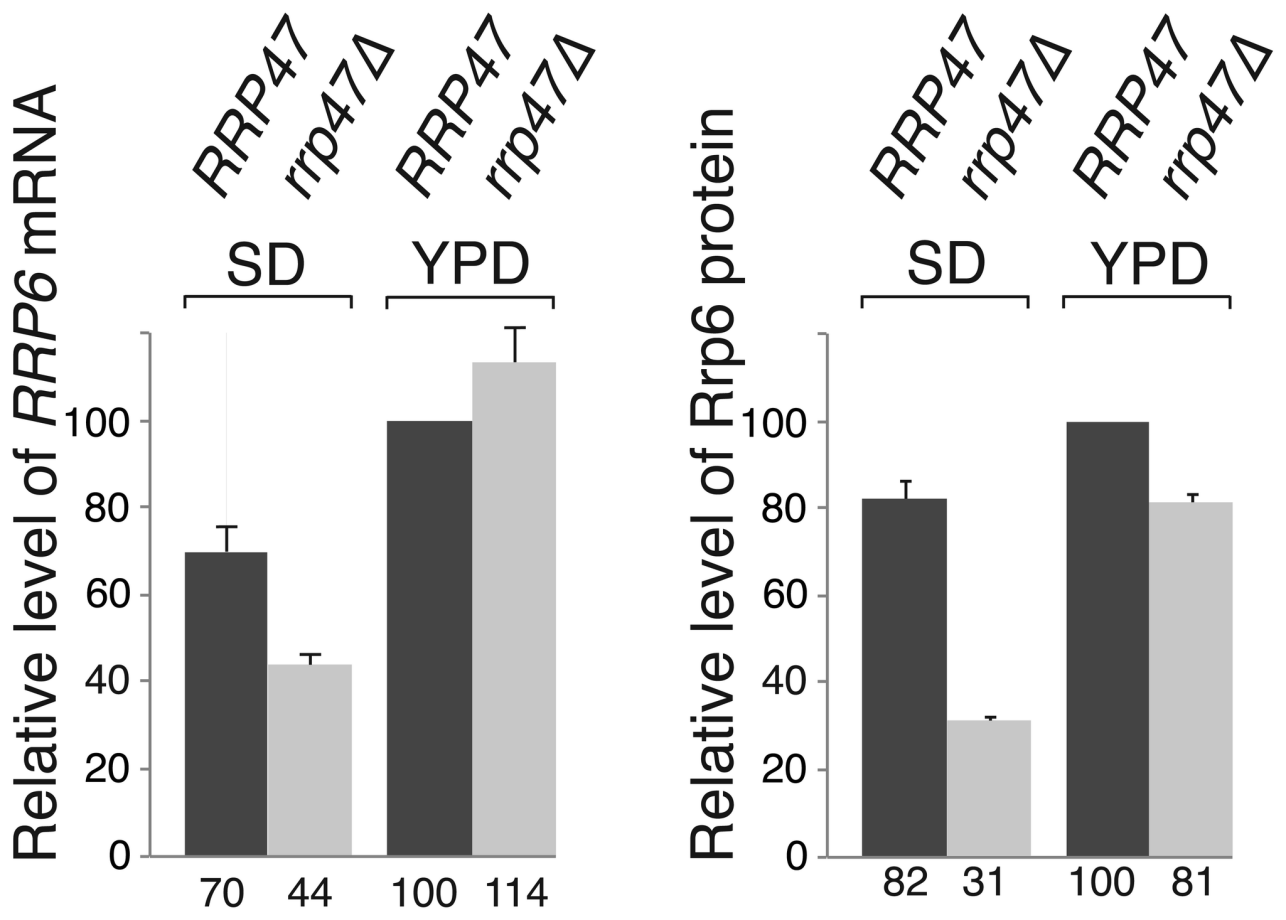
*RRP6* mRNA levels are decreased in the *rrp47∆* mutant. Relative expression levels of *RRP6* mRNA in wild-type and *rrp47∆* mutants during growth in either selective minimal medium (SD) or rich medium (YPD). Expression levels, indicated as percentages, are standardised to the amount in wild-type cells grown in YPD medium. *RRP6* mRNA levels were determined by qRT-PCR and normalised to the *SCR1* RNA. Expression levels of non-tagged Rrp6 protein, determined as in Figure 1, are shown for comparison. Error bars indicate the positive range of the standard error of the mean for each set of values.

The reduction in Rrp47 observed in an *rrp6∆* mutant is principally due to a decrease in protein stability when Rrp6 is not available for interaction [43]. To determine whether Rrp6 is less stable in the absence of Rrp47, cultures of isogenic wild-type and *rrp47∆* strains were treated with the translation inhibitor cycloheximide and the depletion of non-tagged, wild-type Rrp6 was followed by western analyses of cell extracts. Rrp6 levels showed a clear decrease through the 80 minute time-course in the *rrp47∆* mutant, compared to the wild-type strain (Figure 2A,B). Quantitative analyses show that after 60 minutes incubation the Rrp6 levels were reduced by ~ 25 % in the wild-type strain, whereas the reduction was nearly 10-fold in the *rrp47∆* mutant (Figure 2C). The half-life of the Rrp6 protein was estimated to be ~ 25 minutes in the *rrp47∆* mutant and greater than 80 minutes in the wild-type strain (Figure S1). The stability of Rrp6 in the *rrp47∆* mutant relative to the wild-type strain was not further decreased when the cultures were grown in minimal medium (Figures 2 and S1). These results show that Rrp6 is more rapidly degraded in the absence of Rrp47, and suggest that an additional mechanism is responsible for the exacerbated decrease in Rrp6 steady state levels during growth in minimal medium. Quantitative real time PCR (qPCR) analyses revealed that the expression level of *RRP6* mRNA in the *rrp47∆* mutant was reduced to ~ 60% of the level observed in wild-type cells during growth in minimal medium (62.5 %, SEM=3.6 %, n=4), while a slight increase in *RRP6* mRNA levels was observed in the *rrp47∆* mutant during growth in rich medium (114 %, SEM=8.7 %, n=4) (Figure 3). Taken together with the western blotting data, these results are consistent with a decrease in Rrp6 levels in the *rrp47∆* mutant due to a general decrease in Rrp6 protein stability that is augmented by a decrease in *RRP6* mRNA levels during growth in minimal medium.

**Figure 4:**
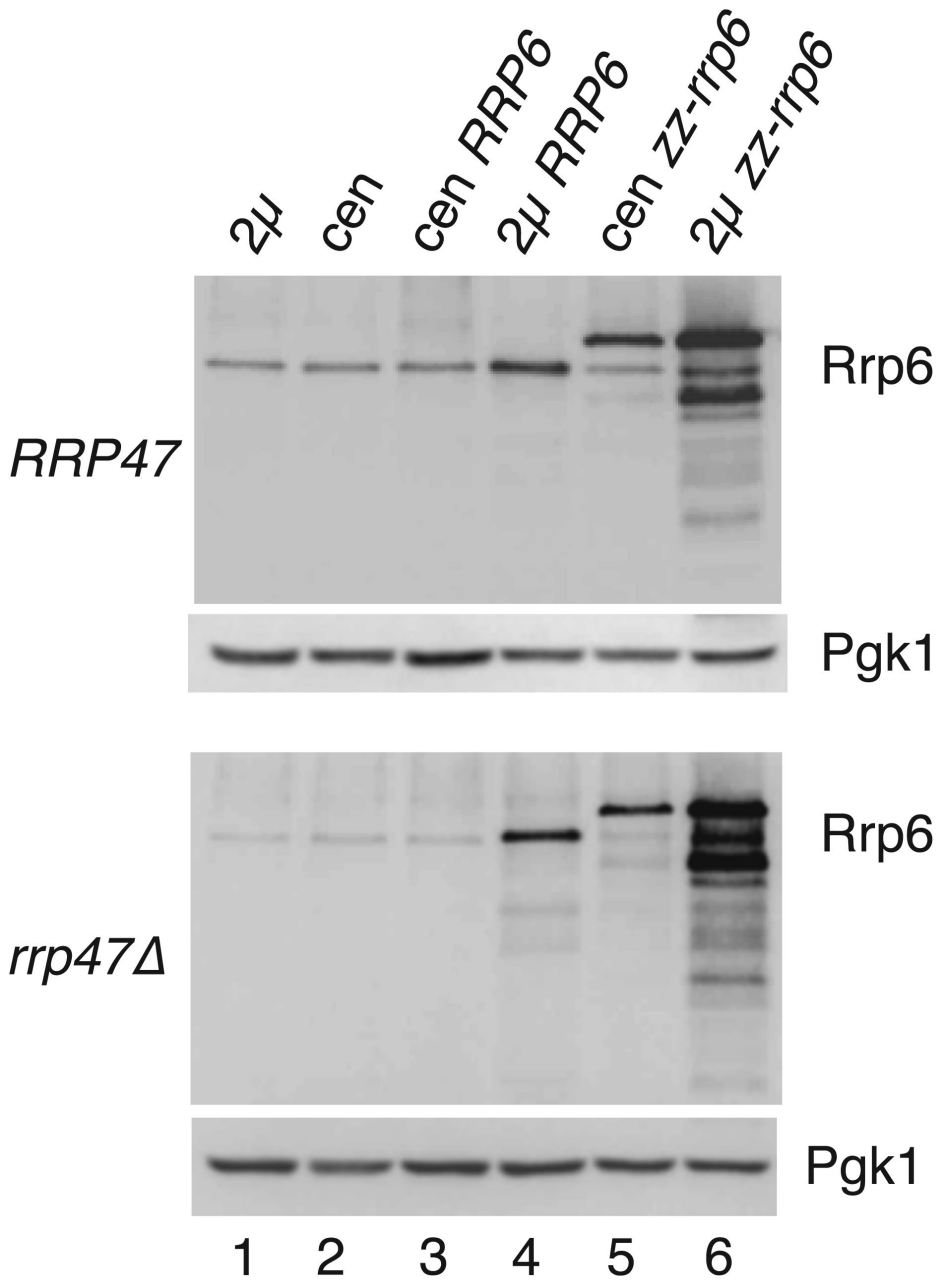
Rrp6 can be overexpressed in wild-type and *rrp47∆* strains. Western analyses of Rrp6 in isogenic wild-type and *rrp47∆* strains that are transformed with *RRP6* expression constructs. Strains were transformed with either the 2 micron (2μ) vector pRS426 (lane 1), the centromeric (cen) plasmid pRS416 (lane 2), an *RRP6* genomic clone in pRS416 (lane 3) or in pRS426 (lane 4), as well as constructs expressing an N-terminal zz fusion of Rrp6 from the *RRP4* promoter in pRS416 (lane 5) or pRS426 (lane 6). Identical blots were analysed for Rrp6 levels using an Rrp6-specific antiserum, followed by analysis of the loading control Pgk1.

To determine whether the expression level of Rrp6 can be increased in the *rrp47∆* mutant by exogenous expression of *RRP6*, isogenic wild-type and *rrp47∆* strains were transformed with low copy, centromeric (cen) or high copy, 2 micron (2μ) plasmids encoding the wild-type *RRP6* gene (see Materials and Methods) and cell extracts from cultures grown in selective minimal medium were analysed by western blotting using an Rrp6-specific antibody [11]. Rrp6 levels were clearly increased in both the wild-type strain and the *rrp47∆* mutant upon transformation with a high copy number plasmid encoding the *RRP6* gene (Figure 4, compare lanes 1-4), with higher expression levels achieved in the *rrp47∆* mutant than are seen in wild-type cells transformed with the vector alone.

We also analysed the relative expression levels of an N-terminal zz-Rrp6 fusion protein [45] expressed from the *RRP4* promoter within either a centromeric or 2μ plasmid. Expression of the zz-Rrp6 fusion protein from a 2μ plasmid was greater than from a centromeric plasmid in both wild-type and *rrp47∆* strains. Thus, although normal Rrp6 expression levels are dependent upon Rrp47 it is nevertheless possible to overexpress Rrp6 in the *rrp47∆* mutant. The lower molecular weight bands observed in Figure 4 upon overexpression of the zz-Rrp6 fusion protein are C-terminal degradation products. These polypeptide fragments are also visible upon expression of the zz-Rrp6 fusion protein from a centromeric plasmid (Figure 4, lane 5) or upon overexpression of non-tagged Rrp6 (Figure 4, lane 4), although at a much reduced level.

### *RRP6* overexpression suppresses RNA defects in *rrp47∆* mutants

**Figure 5:**
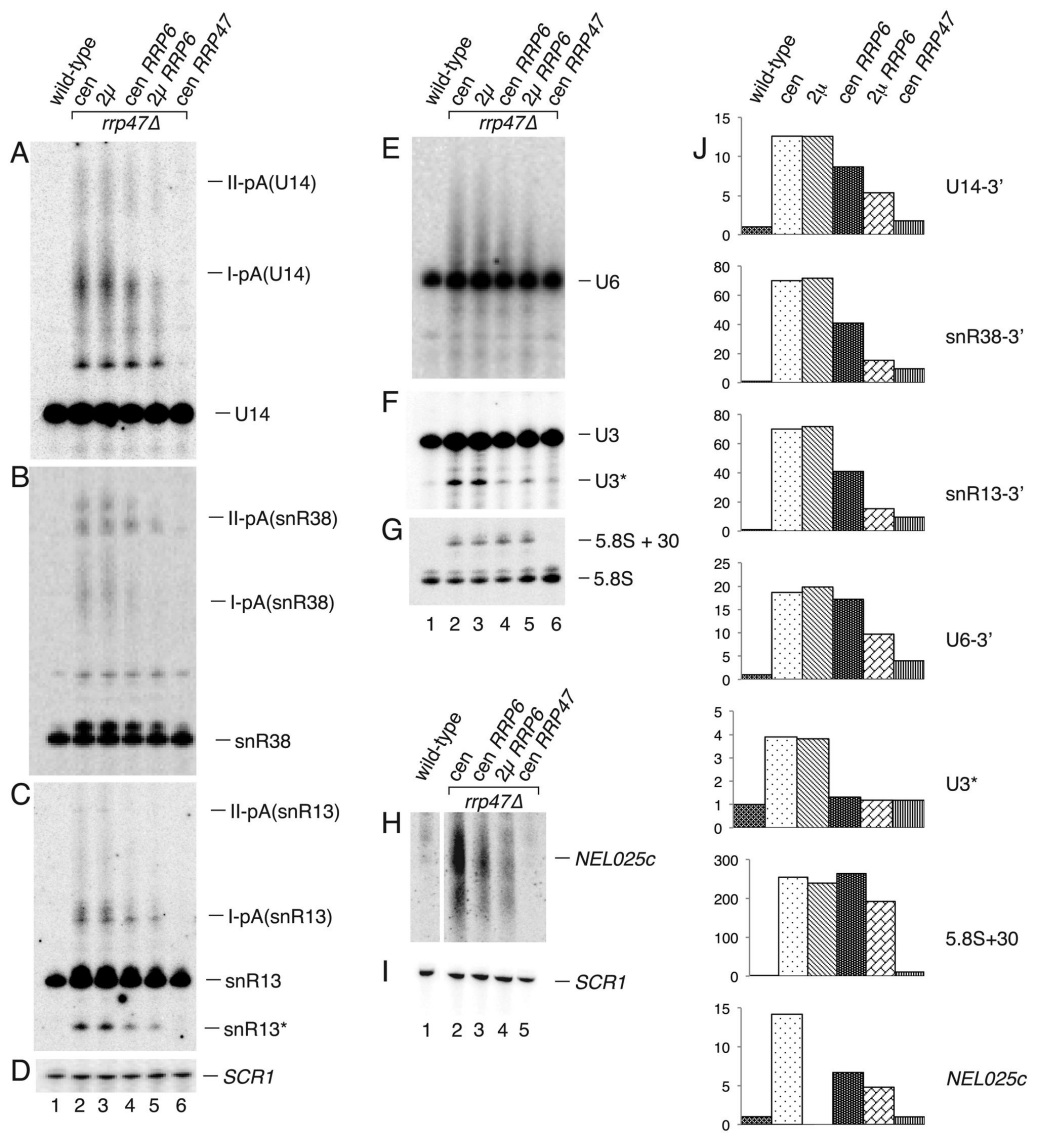
Rrp6 overexpression suppresses RNA phenotypes in *rrp47∆* mutants. Northern analyses of total cellular RNA isolated from a wild-type strain, an isogenic *rrp47∆* mutant and from *rrp47∆* mutants expressing exogenous Rrp6 or Rrp47 from either centromeric (cen) plasmids or 2 micron-based (2μ) constructs. RNA was resolved through 8% denaturing acrylamide gels, transferred to nylon membranes and hybridised with probes complementary to specific RNAs, as follows: (A) U14; (B) snR38; (C) snR13; (D) SCR1; (E) U6; (F) U3; (G) 5.8S; (H) *NEL025c*; (I) *SCR1*. Blots shown in A-G and H-I are from distinct gels. Dispersed bands labelled I-pA and II-pA in panels A-C represent snoRNAs that are polyadenylated after termination at sites I and II, respectively. The bands labelled snR13* and U3* are 5’ truncated forms of snR13 and U3. (J) Quantification of signals for the 3’ extended forms of U14, snR38, snR13, U6 and 5.8S, the truncated U3 RNA and the *NEL025c* mRNA are shown for each strain. Average values of two data sets are normalised to *SCR1* loading controls and expressed relative to the level of the RNA observed in the wild-type strain.

To address whether the RNA processing and degradation defects observed in the *rrp47∆* mutant can be ascribed to an indirect effect of decreased expression of Rrp6, rather than the absence of Rrp47 protein, we performed acrylamide gel northern blot analyses on RNA isolated from *rrp47∆* strains that harboured centromeric and 2μ plasmids expressing Rrp6. It has previously been shown that *rrp6∆* and *rrp47∆* mutants accumulate 3’ extended, polyadenylated forms of snoRNAs [7,11,53]. These extended snoRNA transcripts are thought to arise due to transcription termination at either the downstream proximal site I or the more distal site II, followed by polyadenylation [54]. Exogenous expression of the *RRP6* gene from the 2μ vector substantially reduced the levels of U14, snR38 and snR13 box C/D snoRNAs that are 3’ extended to site I and site II (Figure 5A-C,J) in the *rrp47∆* mutant. Expression of the *RRP6* gene in the *rrp47∆* mutant from a centromeric vector also had a clear effect but the suppression was less marked than when *RRP6* was expressed from the multicopy plasmid (Figure 5A-C, compare lanes 2-5). This suppression in the absence of a clear increase in Rrp6 steady state levels (see Figure 4) may reflect a differential nuclear localization of Rrp6 or an increase in the effective concentration of Rrp6/TRAMP and/or Rrp6/exosome complexes. The accumulation of extended forms of U6 snRNA, truncated forms of U3 and snR13 snoRNAs (denoted as U3* and snR13* in Figure 5) and the *NEL025c* CUT observed in the *rrp47∆* mutant was also suppressed (Figure 5C,E,F,H,J). Notably, some RNAs that accumulate in the *rrp47∆* mutant, such as the “+30” 3’ extended form of 5.8S rRNA were not clearly reduced upon Rrp6 overexpression (Figure 5G,J). This difference may reflect either the extensive degree of secondary structure found at the 3’ end of the 3’ extended 5.8S rRNA [55], its nucleolar localisation or the large degree of flux through the pre-rRNA processing pathway [56]. Increased expression of Rrp6 in the *rrp47∆* mutant did not suppress the RNA processing defects to the extent seen upon transformation with a plasmid bearing the wild-type *RRP47* gene (Figure 5A-G, compare lanes 5 and 6), indicating that Rrp47 has functions in RNA processing and degradation in addition to ensuring adequate expression levels of Rrp6. Overexpression of Rrp6 *per se* is not detrimental to the cell, since no alteration in phenotype was detected upon Rrp6 overexpression in a wild-type strain (unpublished data). These data suggest that the requirement for Rrp47 in box C/D snoRNA maturation, the degradation of CUTs and in RNA surveillance pathways mediated by Rrp6 can be partially attributed to its indirect effect on Rrp6 expression. 

### *RRP6* overexpression suppresses the genetic requirement for *RRP47* expression

**Figure 6:**
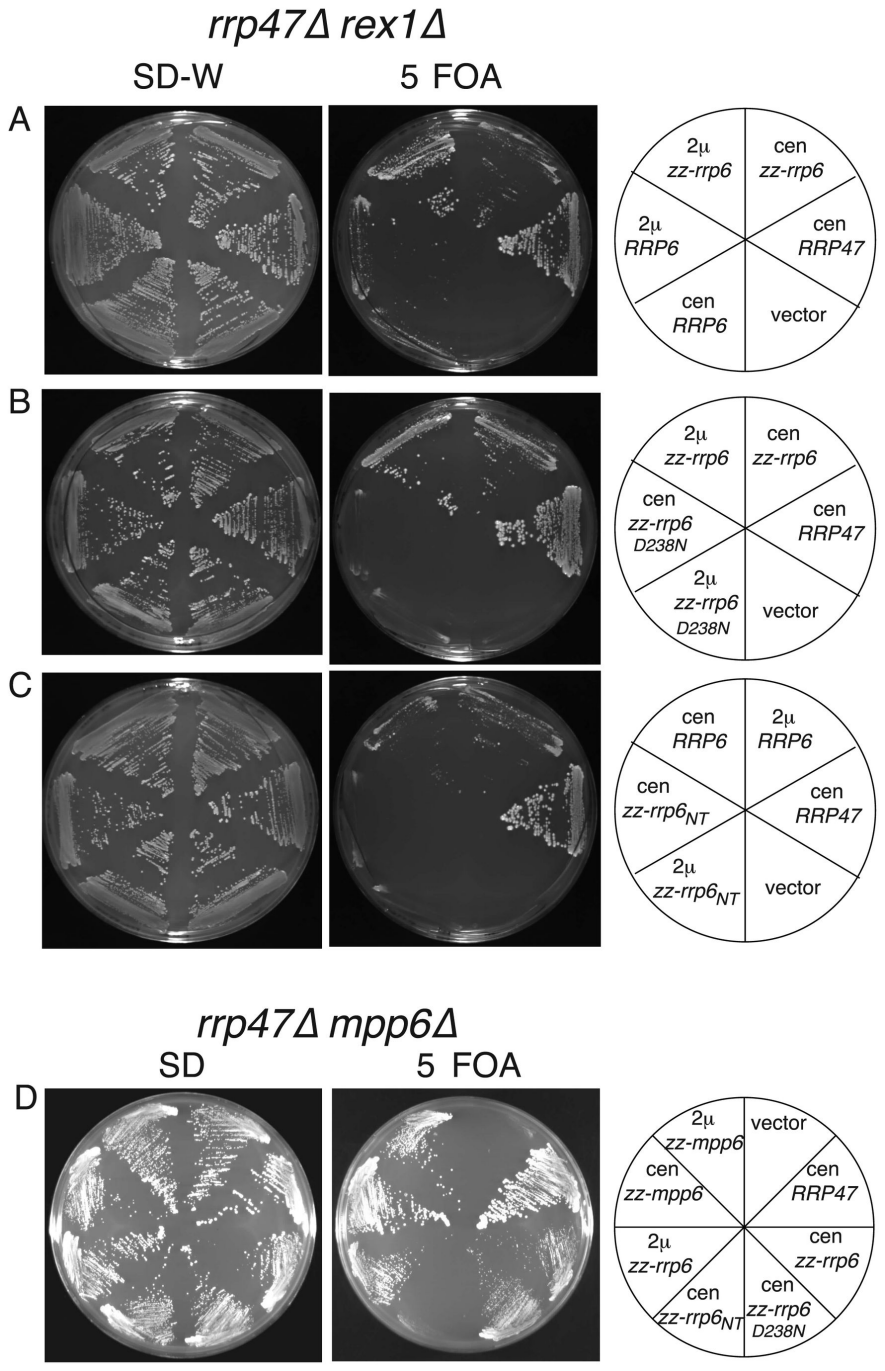
Exogenous expression of Rrp6 complements the synthetic lethality of *rrp47∆ rex1∆* and *rrp47∆ mpp6∆* mutants. Yeast *rrp47∆* *rex1∆* and *rrp47∆ mpp6∆* double mutants bearing plasmids with a *URA3* marker and a wild-type copy of either the *RRP47* (panels A-C) or *MPP6* gene (panel D) were transformed with *RRP6* constructs. Transformants were isolated on selective minimal medium and tested for growth in parallel on permissive minimal medium (left panel) and on medium containing 5 FOA (right panel). Plates were incubated at 30 °C for 3 days. The nature of the expression construct is indicated for each segment on the right.

Yeast *rrp47∆ rex1∆* and *rrp47∆ mpp6∆* double mutants are synthetic lethal [29,33]. To determine whether normal wild-type expression levels of Rrp6 can alleviate the requirement for *RRP47* expression in *rex1∆* or *mpp6∆* mutants, centromeric and 2μ plasmids encoding *rrp6* alleles were transformed into *rrp47∆ rex1∆* and *rrp47∆ mpp6∆* plasmid shuffle strains and the resulting transformants were assayed for growth on medium containing 5 FOA. Isolates were obtained for the *rrp47∆ rex1∆* transformants expressing either wild-type Rrp6 or the zz-Rrp6 fusion protein from both centromeric and 2μ vectors, but not from the vector control, growth being most readily observed upon transformation with the 2μ plasmid encoding the Rrp6 fusion protein (Figure 6A,B). In contrast, no growth was observed upon transformation with constructs encoding the catalytically inactive *rrp6*_*D238N*_ mutant (Figure 6B) or just the N-terminal PMC2NT domain of Rrp6 (*rrp6*_*NT*_) (Figure 6C). These data demonstrate that exogenous expression of Rrp6 suppresses the synthetic lethality of *rrp47∆ rex1∆* mutants, and that the suppression is dependent upon the expression of catalytically active Rrp6. Exogenous expression of the zz-Rrp6 fusion protein also allowed growth of the *rrp47∆ mpp6∆* mutant (Figure 6D). Notably, the *rrp47∆ mpp6∆* double mutant was complemented by expression of the *rrp6*_D238N_ mutant. This suggests that Rrp6 has an important noncatalytic function in cells lacking Mpp6.

**Figure 7:**
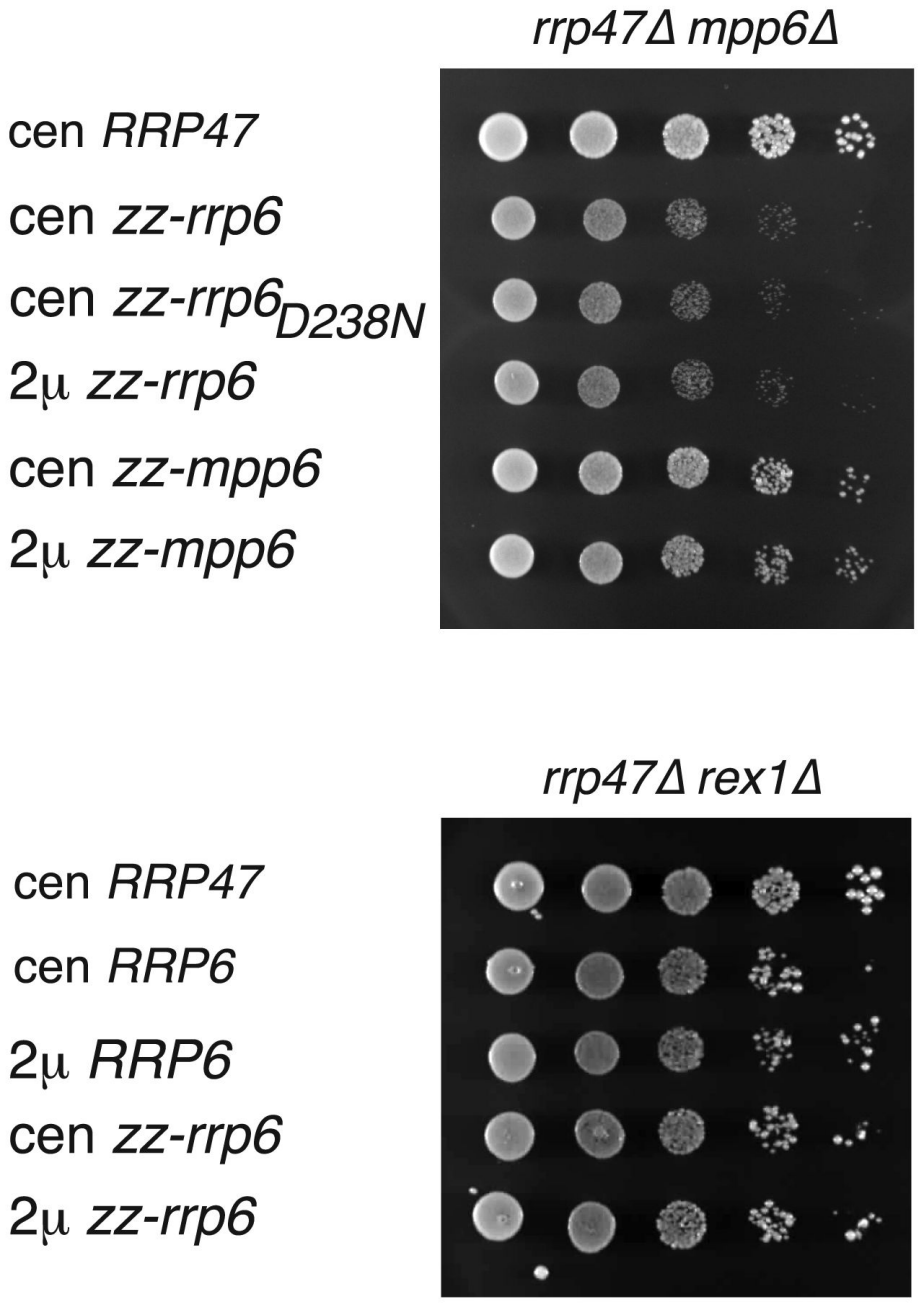
Growth assays of *rrp47∆ rex1∆* and *rrp47∆ mpp6∆* mutants. Spot growth assays of *rrp47∆* *mpp6∆* (upper panel) and *rrp47∆* *rex1∆* (lower panel) double mutant isolates. The complementing construct is indicated on the left. 10-fold serial dilutions of standardised precultures were spotted on to selective solid medium and the plates were incubated at 30 °C. Plates were photographed after incubation for 3 days.

Isolates were recovered from the 5 FOA plates and assayed for growth on solid minimal medium. The growth rates of all the complemented *rrp47∆ rex1∆* double mutants were comparable, whether the plasmids encoded the *RRP6* gene or the *RRP47* gene (Figure 7). In contrast, *rrp47∆ mpp6∆* transformants expressing an increased amount of Rrp6 showed a markedly slow growth phenotype relative to transformants that were complemented by copies of the *RRP47* or *MPP6* gene. This suggests that the *mpp6∆* mutant shows a higher degree of dependence upon expression of the Rrp47 protein for optimal growth than the *rex1∆* mutant.

### Northern analyses of *rrp47∆ rex1∆* and *rrp47∆ mpp6∆* mutants

**Figure 8:**
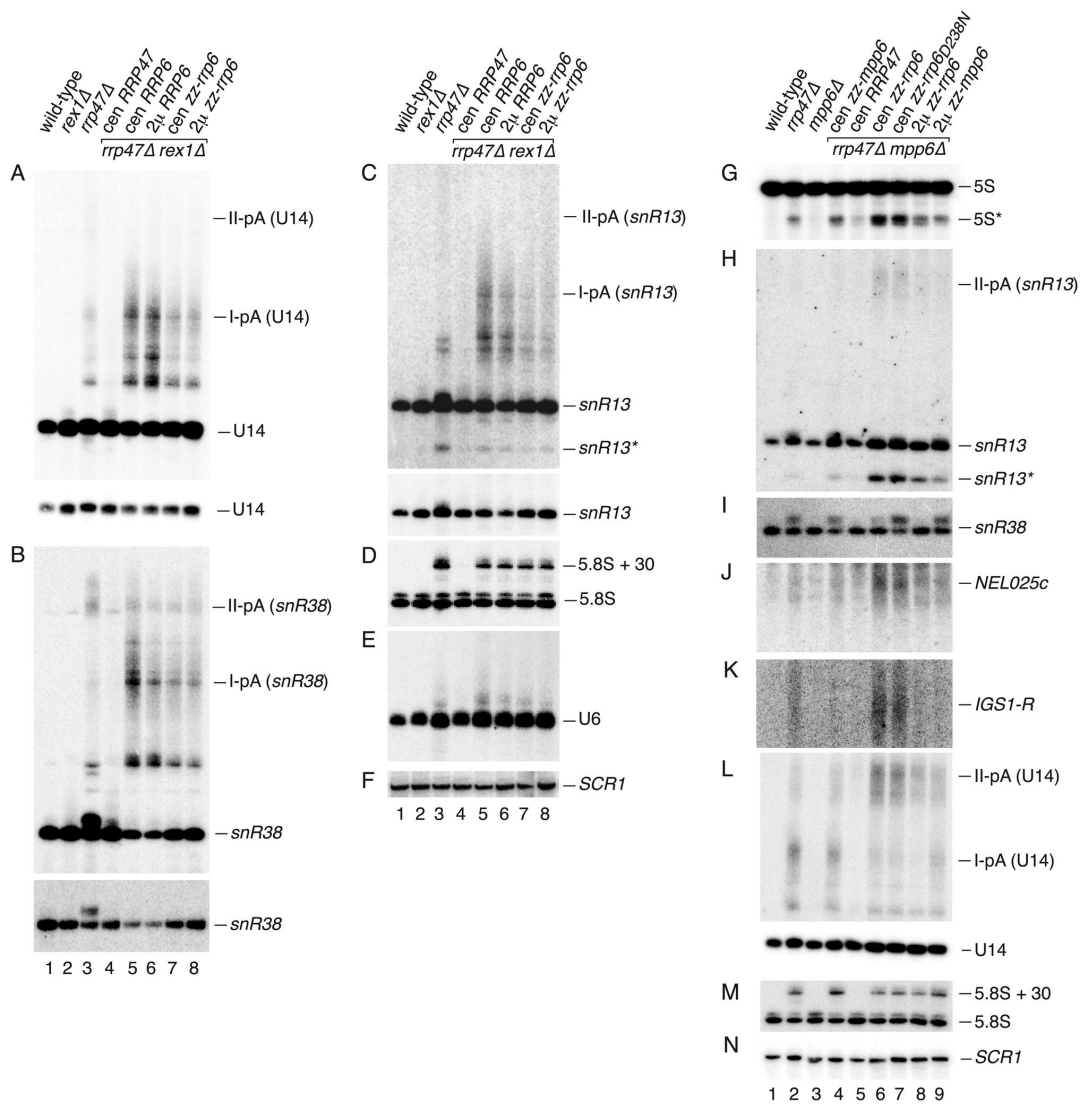
Northern analyses of *rrp47∆ rex1∆* and *rrp47∆ mpp6∆* mutants. Total cellular RNA was isolated from isogenic wild-type, *rex1∆*, *rrp47∆* and *mpp6∆* strains, and from *rrp47∆* *rex1∆* *rrp47∆* or *rrp47∆* *mpp6∆* double mutants that are complemented by centromeric (cen) or 2 micron (2μ) plasmids expressing *RRP47*, *MPP6* or *RRP6* alleles. *RRP6* constructs encoded either non-tagged or zz-tagged fusion proteins. RNA was resolved through 8% denaturing polyacrylamide gels and northern blot analyses performed, using probes complementary to the RNAs indicated on the right of each panel. (A-F) Analysis of *rrp47∆* *rex1∆* mutants. (G-N) Analysis of *rrp47∆* *mpp6∆* mutants. To compare the relative levels of both mature and 3’ extended forms of snoRNAs in the different strains in panels A-C, two images are shown from the same hybridisation. Dispersed bands labelled I-pA and II-pA represent snoRNAs that are polyadenylated after termination at sites I or II, respectively. Bands labelled 5S* and snR13* represent truncated RNAs.

Northern blot analyses were performed on total cellular RNA isolated from the complemented *rrp47∆ rex1∆* and *rrp47∆ mpp6∆* double mutants during growth in minimal medium and compared to RNA from a wild-type strain and from the *rrp47∆, rex1∆* and *mpp6∆* single mutants. The amount of the shorter 3’ extended forms of U14, snR13 and snR38 was dramatically increased in the *rrp47∆ rex1∆* mutant complemented by expression of the *RRP6* gene from the centromeric plasmid, compared to the *rrp47∆* single mutant (labelled I-pA in Figure 8A-C, compare lanes 3 and 5). Complementation of the *rrp47∆ rex1∆* mutant with cen or 2μ plasmids encoding the zz-Rrp6 fusion protein caused a weaker defect in snoRNA 3’ maturation (Figure 8A, lanes 7 and 8). Northern analyses of RNA from multiple *rrp47∆ rex1∆* isolates showed that this effect was reproducible (Figure S2). The milder phenotypes observed in the *rrp47∆ rex1∆* strain expressing the zz-Rrp6 fusion protein presumably reflect the increased expression of this form of Rrp6 (Figure 4). We conclude that the accumulation of 3’ extended snoRNAs in the *rrp47∆ rex1∆* mutants can be alleviated by increased expression of Rrp6. As in the case of the *rrp47∆* single mutant (Figure 5), the extended forms of U6 snRNA were depleted in the *rrp47∆ rex1∆* double mutant upon overexpression of Rrp6 (Figure 8E) and there was no suppression of the 5.8S rRNA processing defect (Figure 8D).

Conditional *rrp47 mpp6* double mutants exhibit defects in the degradation of CUTs and in discard pathways that degrade defective nuclear pre-mRNAs and pre-rRNA fragments [29]. Consistent with this earlier study, northern analyses of RNA from the *rrp47∆ mpp6∆* mutants expressing the zz-Rrp6 fusion protein from a centromeric plasmid revealed defects in the degradation of truncated fragments of 5S rRNA and snR13 (denoted as 5S* and snR13* in Figure 8G and H, respectively) that are presumably targeted to discard pathways [11,57], as well as the accumulation of the *NEL025c* and *IGS1-R* CUTs (Figure 8J,K). In all cases, these RNAs accumulated substantially more in the *rrp47∆ mpp6∆* double mutant expressing zz-Rrp6 from a centromeric plasmid than in the *rrp47∆* or *mpp6∆* single mutants (Figure 8G-K, compare lanes 2,3 and 6). Northern analyses using probes complementary to box C/D snoRNAs revealed that the *rrp47∆ mpp6∆* mutant expressing zz-Rrp6 from a centromeric plasmid also accumulated 3’ extended U14 and snR13 RNAs (Figure 8H,L). In contrast to the *rrp47∆ rex1∆* mutants, however, the longer forms of snR13 and U14 detected in the *rrp47∆ mpp6∆* mutant were extended to site II (labelled II-pA in Figure 8).

The severity of the phenotypes observed in the *rrp47∆ mpp6∆* double mutant expressing exogenously supplied zz-Rrp6 was suppressed upon expression of the protein from a multicopy plasmid (Figure 8G-N, compare lanes 6 and 8). The analysis of RNA from multiple independent isolates showed that this effect is reproducible (Figure S3). Taken together, the data shown in Figure 8 supports a role for Rrp6, directly or indirectly, in the processing or degradation of RNAs that accumulate in the *rrp47∆ rex1∆* or *rrp47∆ mpp6∆* mutants. Yeast snoRNA maturation is dependent upon the exonuclease activity of Rrp6 in the case of the *rrp47∆ rex1∆* mutant, since no complementation was observed for the catalytically inactive *rrp6*_*D238N*_ mutant (Figure 6B). In contrast, the *rrp47∆ mpp6∆* mutant could be complemented by expression of the *rrp6*_D238N_ mutant and the severity and nature of the RNA phenotypes seen upon complementation were generally indistinguishable to that seen upon expression of the wild-type protein (Figure 8G-N, compare lanes 6 and 7). These observations provide support for an important non-catalytic role of Rrp6 in RNA surveillance and degradation pathways that has been noted in previous studies [58]. The *rrp6*_*D238N*_ mutant was not, however, able to process the short 3’ extensions of snoRNAs (resolved from the mature RNA well in the case of snR38 in Figure 8I, compare lanes 6 and 7) that arise through the addition of short oligoadenylate tails [54].

**Figure 9:**
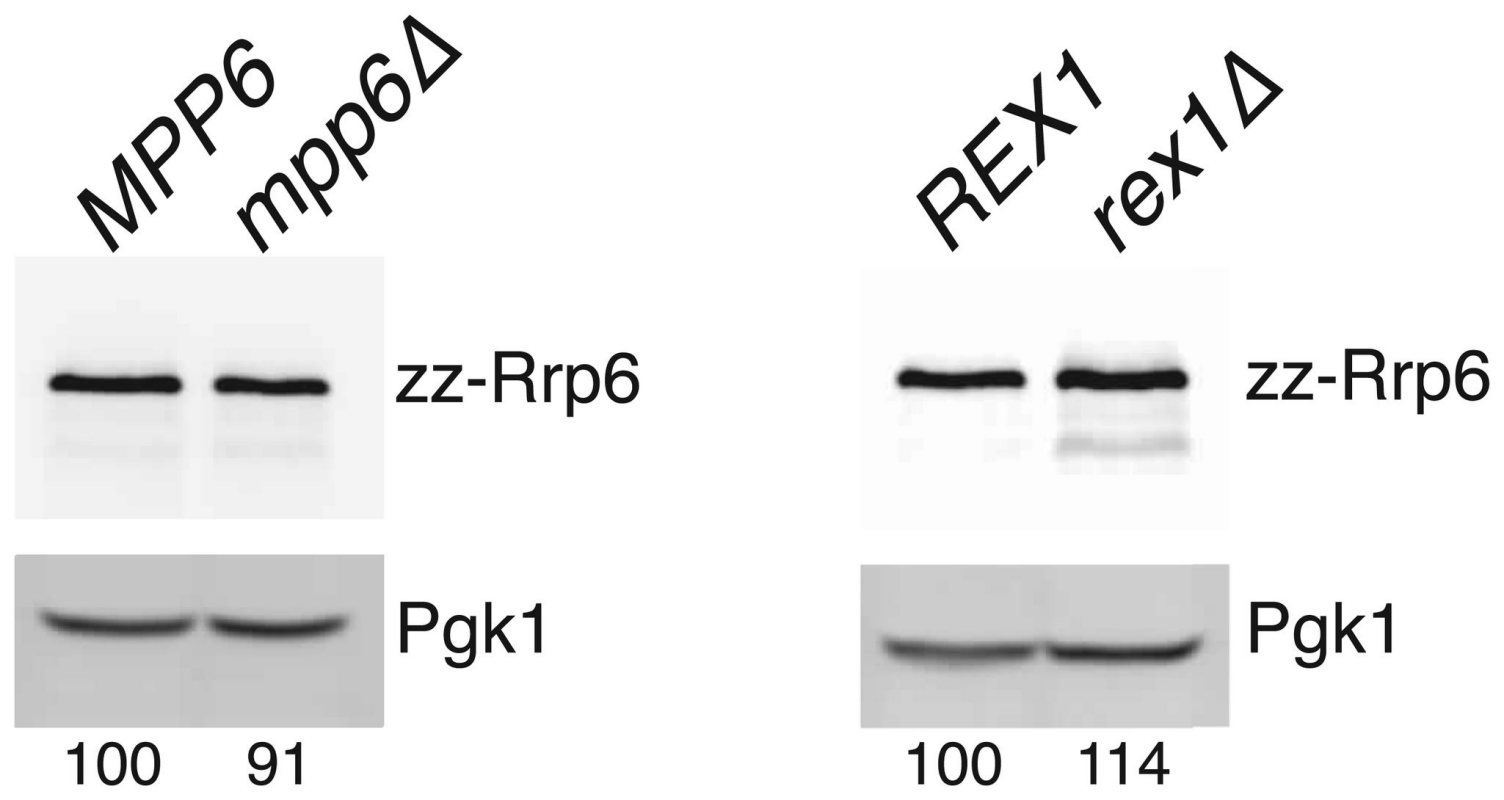
Rrp6 levels are not altered in *mpp6∆* or *rex1∆* mutants. Western analyses were performed on extracts from strains that either carry a wild-type or a deletion allele of the *MPP6* or *REX1* gene and that express the zz-Rrp6 fusion protein. Blots were successively incubated with the PAP antibody and antibody specific to the Pgk1 protein. Expression levels of zz-Rrp6 in the *mpp6∆* and *rex1∆* mutants, relative to the level observed in the corresponding wild-type strain, are given at the bottom of the figure and are the average of three independent biological replicates.

Given that the exonuclease activity of Rrp6 is redundant with activities dependent upon either Mpp6 or Rex1 [44](Figure 6), we hypothesised that the expression of Rrp6 may be increased when Mpp6- or Rex1-dependent pathways are blocked. To address this, we determined the relative levels of Rrp6 in extracts of isogenic strains that carry either a wild-type or null allele of the *MPP6* or *REX1* gene. Western analyses of cultures grown in minimal medium showed that Rrp6 expression levels are not markedly altered in the presence or absence of Mpp6 or Rex1 (Figure 9). Thus, Rrp6 expression levels are responsive to the availability of its interacting protein Rrp47 but not the status of redundant Mpp6- or Rex1-dependent processing or degradation pathways.

## Discussion

The yeast protein Rrp47 was identified 10 years ago as an exosome-associated protein that is functionally linked to the Rrp6 exonuclease [11,33,59]. The exact molecular function(s) of this protein has, however, remained largely elusive. We have recently shown that the stability of Rrp47 is drastically reduced in the absence of Rrp6 [43]. Here we demonstrate that Rrp6 protein levels are reduced in the absence of Rrp47, and that this effect is reinforced when strains are grown in minimal medium rather than rich medium. Down-regulation of Rrp6 expression occurs both at the level of protein stability and at the *RRP6* transcript level. Strikingly, restoration of Rrp6 expression in an *rrp47∆* mutant to wild-type levels is largely sufficient to compensate for the lack of Rrp47, suppressing RNA processing and turnover defects observed in the *rrp47∆* mutant and complementing the synthetic lethal growth phenotypes of *rrp47∆ rex1∆* and *rrp47∆ mpp6∆* double mutants. This demonstrates that an important function of Rrp47 is to facilitate a critical expression level of Rrp6. Similar findings have been independently reported recently [60]. In contrast to the study by Stuparevic et al., we did not observe a complete depletion of Rrp6 in the absence of Rrp47. Our findings are more consistent with observed differences in the growth phenotypes and genetic interactions of *rrp47∆* and *rrp6∆* mutants [11].

The Rrp6 and Rrp47 proteins interact directly with one another [27]. The mutual stabilisation of two interacting proteins has the consequence of limiting the expression of the constituent proteins to functional, assembled complexes and suppressing the potential titration of substrates or factors by the one or other subunit. In the case of Rrp47 and Rrp6, this would limit the expression of the proteins to their site of assembly and functional location in the cell nucleus [43]. Consistent with a key function of Rrp47 being its ability to facilitate normal Rrp6 expression levels, the Sas10/C1D domain of Rrp47 that is required for the interaction with Rrp6 is sufficient for the function of the protein *in vivo* [28]. Notwithstanding this impact of Rrp47 on Rrp6 expression levels, Rrp47 performs additional functions as part of the Rrp6/Rrp47 complex that contribute to RNA processing and degradation and which involve the C-terminal region of the protein [28,44]. This is underlined by the observation that expression of exogenous Rrp6 protein restored Rrp6 levels but did not suppress the defects in RNA processing and degradation in the *rrp47∆* single mutant or either double mutant as efficiently as expression of the *RRP47* gene.

The ability of increased Rrp6 expression to suppress the requirement for Rrp47 facilitated the isolation of viable *rrp47∆ rex1∆* and *rrp47∆ mpp6∆* strains, and the analyses of the complemented strains provided some insight into the molecular basis of the synthetic lethal relationship between *rrp47∆* mutations and *rex1∆* or *mpp6∆* alleles. Northern analyses of the *RRP6*-complemented *rrp47∆ rex1∆* double mutants revealed an accumulation of 3’ extended box C/D snoRNAs that was suppressed upon increased expression of Rrp6. Furthermore, the exonuclease activity of Rrp6 is required for complementation of the *rrp47∆ rex1∆* mutant. We recently reported that segregation of Rrp47 from catalytically active form of Rrp6 in the *rex1∆* mutant causes a block in the 3’ end maturation of box C/D snoRNAs [44]. This strongly suggests that Rrp6 and Rex1 have a redundant function in snoRNA processing that cannot be carried out by other cellular exonucleases. There are no additional detectable snoRNA processing intermediates in the *rrp47∆* mutant upon loss of Rex1, suggesting that the redundancy between Rrp6 and Rex1 does not stem from a cooperative pathway. This is further supported by the lack of data supporting a physical interaction between these two proteins or with a mutual partner. Rather, the redundancy of processing activities in snoRNA maturation appears to reflect genetic buffering. Rex1 is a member of a family of related exonucleases in yeast that remain relatively poorly characterised [32,61]. It will be of interest to address whether the substrate specificity or availability of Rex1 is regulated by an associated protein in a manner similar to the effect of Rrp47 on Rrp6.

In contrast to the specific snoRNA processing defect observed in the *rrp47∆ rex1∆* mutant, a set of RNAs accumulated in the *rrp47∆ mpp6∆* mutant that are normally destined for rapid degradation. These phenotypes were also suppressed upon increased expression of Rrp6, indicative of a functional redundancy between the Rrp6/Rrp47 complex and an Mpp6-dependent activity in RNA discard pathways and the degradation of CUTs [29]. Interestingly, exogenous expression of either the *rrp6-1* allele or the wild-type *RRP6* gene had comparable effects on the growth of the *rrp47∆ mpp6∆* double mutant and the viable transformants were compromised to a similar degree in their ability to degrade truncated stable RNAs, CUTs or extended snoRNA transcripts. These observations are consistent with a previous study proposing that Rrp6 has an important noncatalytic role in RNA surveillance [58]. One noncatalytic mechanism by which Rrp6 might promote RNA surveillance is through its interaction with the exosome complex, which promotes channelling of RNA substrates through the core to Rrp44 [19,26]. However, *rrp6-1 mpp6∆* double mutants are nonviable [44], suggesting that at least one essential biological process is dependent upon the catalytic activity of either Rrp6 or an Mpp6-dependent activity. This most likely reflects the substrate overlap observed for Rrp6 and Rrp44 [3,31]. The molecular function of Mpp6 remains unclear. The human Mpp6 protein physically interacts with homologues of Mtr4 and the Rrp47/Rrp6 complex [62] and contacts the exosome independently of Rrp44 [63], while yeast *mpp6∆* mutants show strong genetic interactions with mutants lacking the TRAMP component Air1 [29,64], as well as mutations of the Rrp6/Rrp47 complex. One possibility is that Mpp6 might act to functionally couple the TRAMP and Rrp6/exosome complexes [30].

Studies on the prokaryotic exoribonucleases RNase R and RNase II have revealed changes in the expression levels of these enzymes upon changes in nutrient availability or other forms of stress [38,39]. The wide-ranging impact on diverse aspects of RNA metabolism that are seen for mutants of the exosome complex, or the 5’→3’ exoribonucleases Xrn1 and Rat1, suggests that modulation of the expression of eukaryotic exoribonucleases may orchestrate similar changes in the transcriptome associated with cellular responses to physiological signals. More detailed analyses of the expression levels of eukaryotic ribonucleases in response to altered growth conditions may be a fruitful area of future research.

## Supporting Information

Figure S1 **Rrp6 stability is decreased in *rrp47∆* mutants.** Western analyses were performed on cell extracts from wild-type strains and *rrp47∆* mutants during growth in rich medium (YPD) or minimal medium (SD) following treatment with cycloheximide (CHX) for the times indicated. The amount of Rrp6 detected was normalised to the level of endogenous Pgk1 protein and expressed as a percentage of the protein present in the cell lysates as a function of time.
(TIF)

Figure S2 **RNA analyses of independent *rrp47∆rex1∆* isolates.** Acrylamide gel Northern analyses were performed on total cellular RNA from independent isolates of the *rrp47∆* *rex1∆* mutant that harbor either the centromeric *RRP6* plasmid (lanes 1-3), the 2μ *RRP6* plasmid (lanes 4-6), a centromeric plasmid encoding the zz-Rrp6 fusion protein (lanes 7-9) or a 2μ plasmid encoding zz-Rrp6 (lanes 10-12). The blot was successively hybridised with probes complementary to snR13, U14, 5.8S rRNA and SCR1.
(TIF)

Figure S3 **RNA analyses of independent *rrp47∆ mpp6∆* isolates.** Acrylamide gel Northern analyses of total cellular RNA from independent isolates of the *rrp47∆* *mpp6∆* mutant harbouring centromeric plasmids encoding Rrp47 (cen *RRP47*), the zz-Mpp6 fusion (cen *zz-mpp6*), the zz-Rrp6 fusion (cen *zz-rrp6*) or the catalytically inactive *rrp6* mutant (cen *zz-rrp6*_*D238N*_), or 2μ plasmids encoding the zz-Rrp6 fusion (2μ *zz-rrp6*) or the zz-Mpp6 fusion (2μ *zz-mpp6*). RNA from four sets of isolates (labelled sets 1-4: lanes 1-6, 7-12, 13-18 and 19-24, respectively) was analysed by hybridisation using probes complementary to the RNAs indicated on the right.
(TIF)
