# Supplementary figures and images for: The Exosome Cofactor Rrp47 Is Critical for the Stability and Normal Expression of Its Associated Exoribonuclease Rrp6 in Saccharomyces cerevisiae
Source: PLoS One. 2013 Nov 5;8(11):e80752. doi: 10.1371/journal.pone.0080752 (PMC3818262; doi:10.1371/journal.pone.0080752)

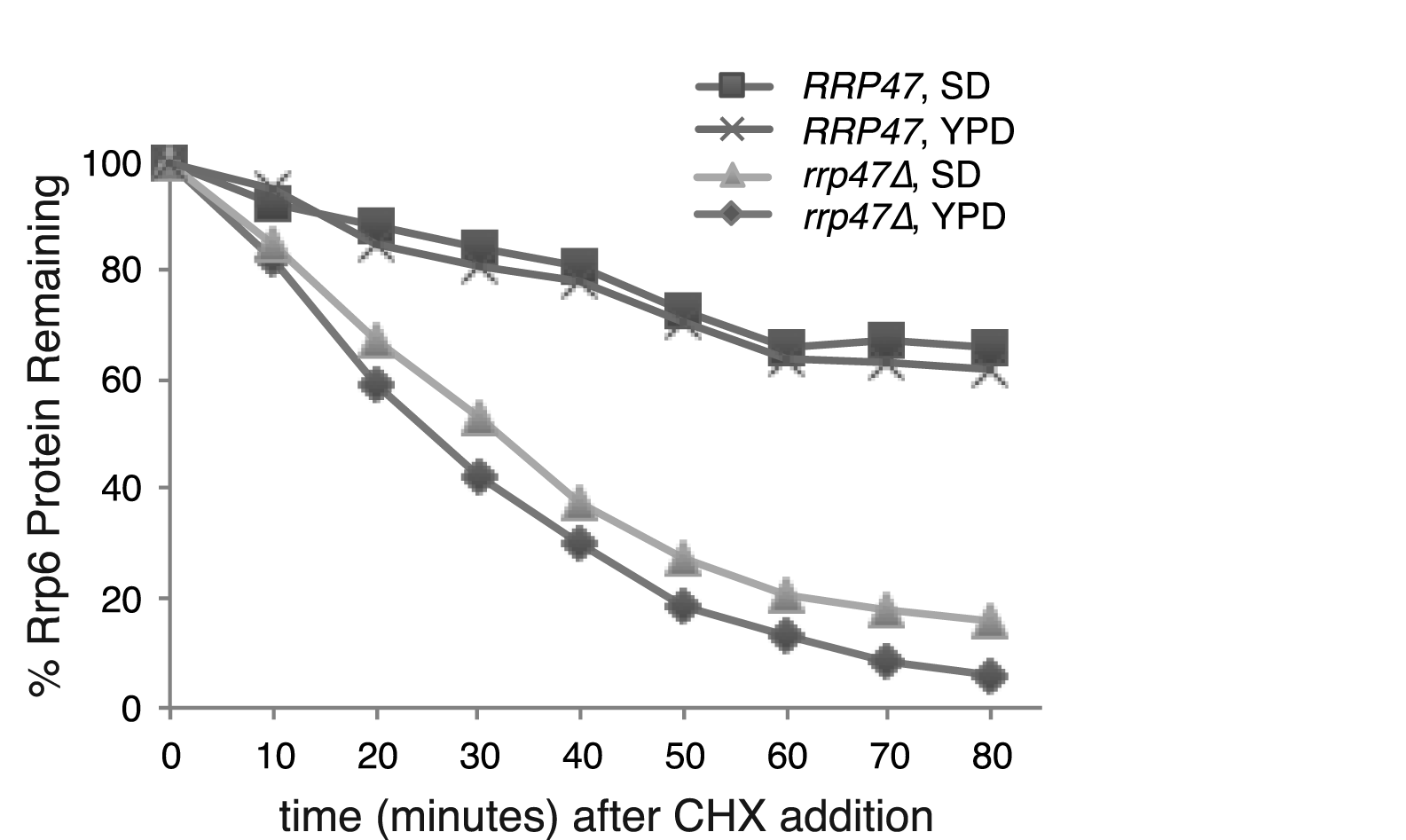

Supplement: Figure S1 — Rrp6 stability is decreased in rrp47∆ mutants. Western analyses were performed on cell extracts from wild-type strains and rrp47∆ mutants during growth in rich medium (YPD) or minimal medium (SD) following treatment with cycloheximide (CHX) for the times indicated. The amount of Rrp6 detected was normalised to the level of endogenous Pgk1 protein and expressed as a percentage of the protein present in the cell lysates as a function of time. (TIF) [file pone.0080752.s001.tif]

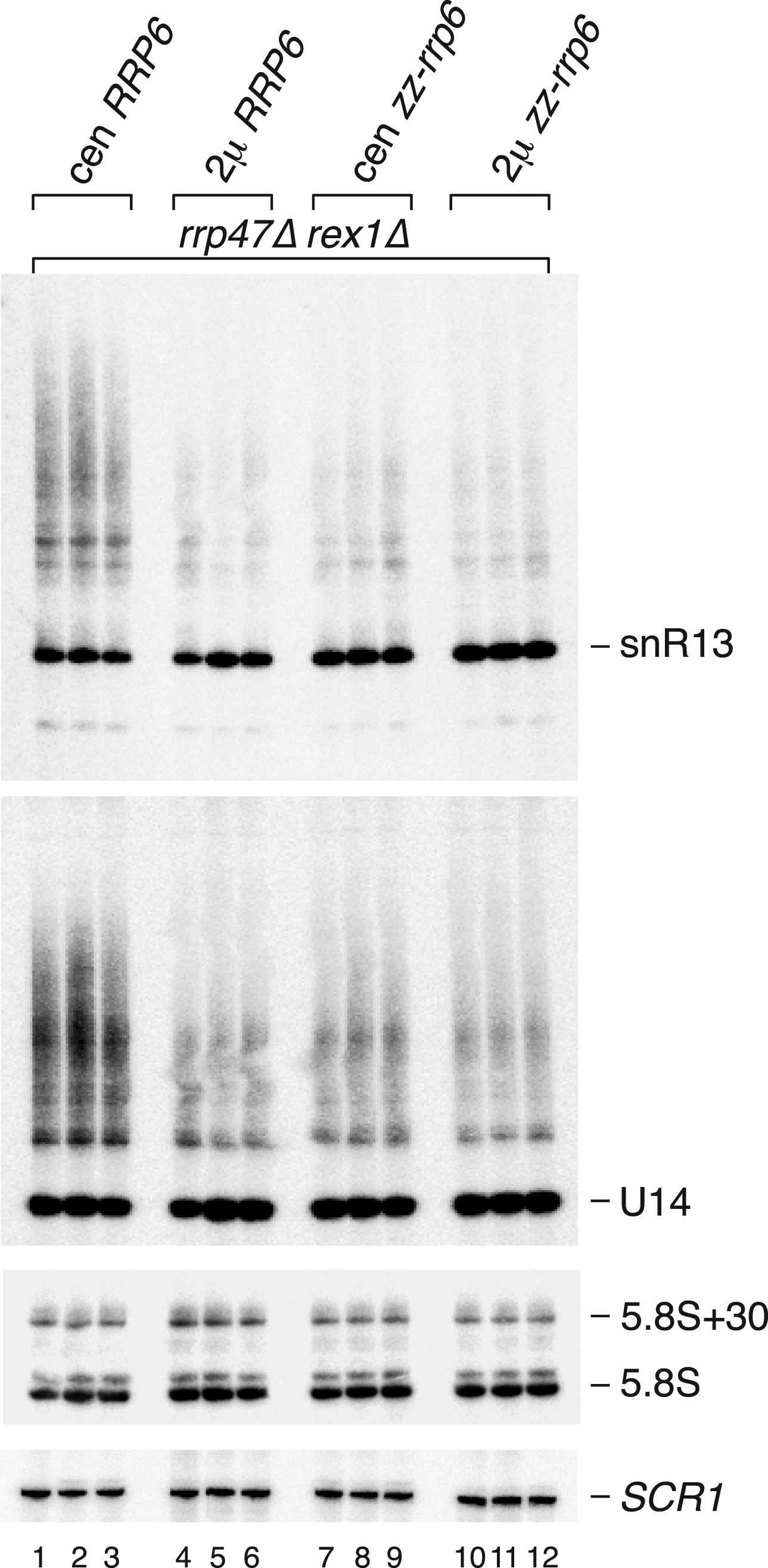

Supplement: Figure S2 — RNA analyses of independent rrp47∆rex1∆ isolates. Acrylamide gel Northern analyses were performed on total cellular RNA from independent isolates of the rrp47∆ rex1∆ mutant that harbor either the centromeric RRP6 plasmid (lanes 1-3), the 2μ RRP6 plasmid (lanes 4-6), a centromeric plasmid encoding the zz-Rrp6 fusion protein (lanes 7-9) or a 2μ plasmid encoding zz-Rrp6 (lanes 10-12). The blot was successively hybridised with probes complementary to snR13, U14, 5.8S rRNA and SCR1. (TIF) [file pone.0080752.s002.tif]

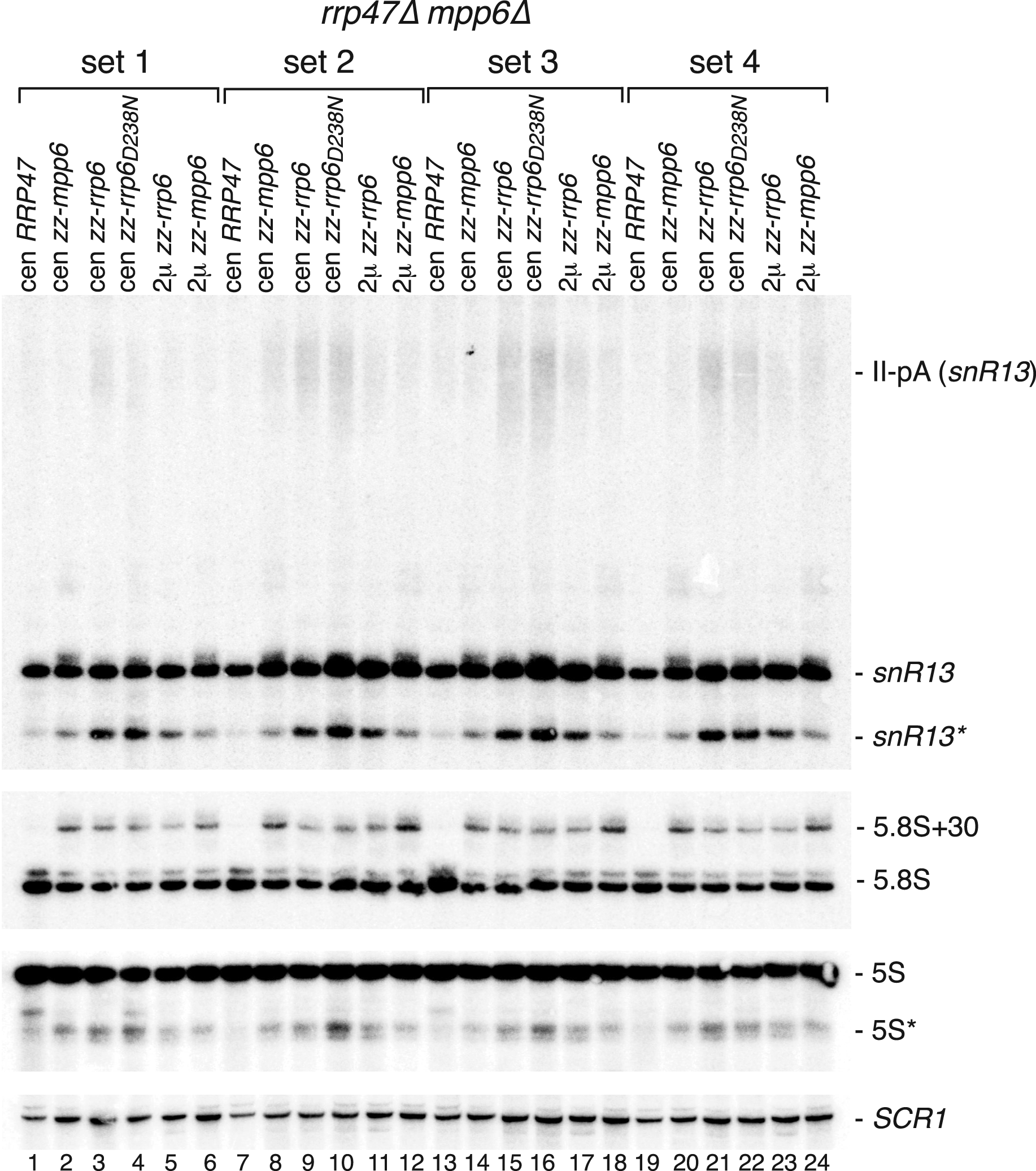

Supplement: Figure S3 — RNA analyses of independent rrp47∆ mpp6∆ isolates. Acrylamide gel Northern analyses of total cellular RNA from independent isolates of the rrp47∆ mpp6∆ mutant harbouring centromeric plasmids encoding Rrp47 (cen RRP47), the zz-Mpp6 fusion (cen zz-mpp6), the zz-Rrp6 fusion (cen zz-rrp6) or the catalytically inactive rrp6 mutant (cen zz-rrp6 D238N), or 2μ plasmids encoding the zz-Rrp6 fusion (2μ zz-rrp6) or the zz-Mpp6 fusion (2μ zz-mpp6). RNA from four sets of isolates (labelled sets 1-4: lanes 1-6, 7-12, 13-18 and 19-24, respectively) was analysed by hybridisation using probes complementary to the RNAs indicated on the right. (TIF) [file pone.0080752.s003.tif]
